# The Four-Cluster Spectrum of Mind-Body Interrelationships: An Integrative Model

**DOI:** 10.3389/fpsyt.2019.00039

**Published:** 2019-03-01

**Authors:** Yacov Ezra, Oded Hammerman, Golan Shahar

**Affiliations:** ^1^Faculty of Medical Sciences, Ben Gurion University of the Negev, Beersheba, Israel; ^2^Department of Neurology, Soroka University Medical Center, Beersheba, Israel; ^3^Psychology Department, Ben Gurion University of the Negev, Beersheba, Israel

**Keywords:** bio-psycho-social model, medical psychology, psychosomatic medicine, mind-body medicine, functional somatic syndromes, hypnosis, cognitive behavioral therapy

## Abstract

Despite the shift toward a biopsychosocial paradigm of medicine, many physicians and mental health professionals (MHPs) find it difficult to treat patients with psycho-somatic disorders. This situation is particularly troublesome due to the high prevalence of these conditions. Although progress has been made over the last few decades in understanding mechanisms underlying the mind-body relationship, disparities remain between research and its clinical implementation. One possible reason for this is the lack of a comprehensive, agreed-upon model that incorporates a biopsychosocial framework and is rooted in an understanding of the various psychobiological pathways. Such a model would enable better communication between physicians and MHPs, allowing them to provide coordinated, stratified treatment. In this paper, four archetypal case studies, together with standard care options are presented to illustrate the current state of affairs. A four-tiered conceptual model of mind-body interrelationships based on pathophysiological and psychopathological mechanisms is suggested to help optimize the treatment of somatic complaints. This Four-Cluster model consists of: (1) *Organic Conditions:* Structural, or degenerative processes that can affect mood and psychological responses but are not clearly exacerbated by stress. (2) *Stress Exacerbated Diseases:* Biological disorders with a distinct pathophysiology, such as inflammatory or autoimmune diseases, whose progression is clearly exacerbated by stress. (3) *Functional Somatic Syndromes*: Conditions wherein heightened sensitivity to stimuli together with hyper-reactivity of the autonomic system form a “vicious cycle” of mutually enhancing learning processes. These processes involve biological mechanisms, such as central sensitization and psychological mechanisms such as catastrophization and selective attention. (4) *Conversion Disorder:* Physical manifestations of psychological distress, expressed somatically. Symptoms are solely an expression of problems in patients' psychic functioning and are not caused by biological pathology. Finally, suggested management of the aforementioned case studies is presented through the lens of the Four-Cluster model and a proposed integration of our model with existing theories is discussed. As it is rooted in an understanding of psychobiological pathways of illness, the proposed model enables a new way to discern which form of mind-body interaction is manifesting in different diseases and proposes a way to coordinate treatment plans accordingly, to enhance the accuracy and efficacy of care.

## Background

The interrelatedness of mind and body has been known to physicians and patients for millennia. Despite this, treating conditions that involve complicated iterations of this relationship has proved difficult and continues to constitute a barrier to the effective care of many patients in the medical system. One contributing factor is that many physicians and mental health professionals (MHPs) still have a dichotomous view of medical conditions as belonging to either pathologies of the “body” or of the “mind” (1). In the past hundred years, many theories have been proposed regarding psychosomatic disorders, with varying levels of mind-body integration. Freudian theory may be considered one of the most influential early attempts at this integration. Freud recognized the difference between four types of somatic illness: (1) hysterical conversion, (2) somatic symptoms of actual neurosis, (3) hypochondria, and (4) organic illness. In all four types, Freud relates pathological body states to pathologies of the mind, so that even organic illnesses are seen as caused by problematic ego-organization or psychic trauma. In line with Freud's work, and in opposition to more medically-oriented approaches, additional psychoanalytic theories have tended to view all types of somatic illness as beginning with problems in the patient's psychic functioning (2).

A new direction was undertaken in the 1930s and 1940s, when Franz Alexander and Flanders Dunbar established the Journal of Psychosomatic Research and discussed ways in which physiological systems mediate between repressed emotions and particular organ pathologies. This contributed to the idea that specific psychopathological profiles influence immunologic, endocrine and neurologic systems, thereby creating certain “psychosomatic” illnesses such as ulcers, hyperthyroidism, arthritis, colitis, and dermatitis (3). Over time, the psychosomatic movement was challenged from different directions. Psychoanalytic theoreticians criticized the psychoanalytic theories (2), while medical scientists noted problems with the research methodologies and the lack of sufficient evidence linking psychological traits to specific illnesses (3, 4). Another important step in the history of mind-body relations was the development of the Bio-Psycho-Social model in the 1970s. In a classic article, George Engel challenged the prevalent Bio-Medical paradigm for looking at medical illness through an extremely narrow biological lens, while ignoring the psycho-social parameters that influence disease (5). In conjunction with the ideas of other prominent scientists, such as Walter Cannon and Hans Selye, Engel sought to look at the aggregate of biological, social, and psychological factors that influence all forms of illness, without distinguishing between psychosomatic and organic illness. He then called on physicians to address the psycho-social needs of their patients from a more humanistic point of view (3, 5). Lastly, in the 1990s, the burgeoning field of Psycho-Neuro-Immunology (PNI) began to describe specific biological mechanisms by means of which the stress response can influence the immune and endocrine systems. PNI studies have shown how stress mediates diseases such as asthma, neurogenic inflammation, autoimmune diseases among others (6–9).

Despite these advancements, a fully integrated model of the ways in which mind and body interrelate remains elusive. Different theories, stemming from various disciplines and viewpoints abound, while the lack of a comprehensive model for integrating these perspectives prevents many of these ideas from becoming integrated into daily clinical practice (10, 11). This lack is reflected in many cases, where physicians diagnose and treat patients' medical complaints while MHPs simultaneously treat their emotional well-being without ever communicating with one another. Even when they correspond with one another, clinicians often do not speak the same “language.” The purpose of a mind-body model would be to integrate different theoretical perspectives, creating a joint paradigm for physicians and MHPs to better understand, communicate and treat complex cases. We would like to illustrate the way such a model could help manage day-to-day difficulties in medical practice by means of the following four imaginary cases. These cases are amalgams and do not represent actual patients. Their purpose is to concretize the difficulties facing clinicians in their daily practice and elucidate our theoretical model.

## Illustrative Vignettes

Case # 1: A 70-year-old man has colon cancer which has metastasized to the liver. He is clinically depressed and low-functioning, with little motivation to continue treatment. His oncologist attempts to convince him to continue taking his medication, without success.
Case # 2: A 25-year-old woman was diagnosed with multiple sclerosis (MS) at the age of 23. Since then, she has begun studying at university in an extremely competitive program and is currently experiencing a great deal of stress related to her studies. At the same time, her MS attacks have become more frequent. The young woman feels that there is a connection between her studies and the attacks; however, her neurologist's response is to change her prophylactic medication to a stronger or higher dose.
Case # 3: A 50-year-old man recently suffered a myocardial infarction. He experiences recurrent chest pains, with repeated admissions to the emergency room. Over the course of treatment, a complete cardiac workup including ECG, scintigraphy, and coronary catheterization are all normal. His cardiologist tells him that it must be anxiety. However, the patient is insulted by this and seeks another physician to continue checking for a physical problem.
Case # 4: A 42-year-old man arrived at the emergency department with a sudden speech disturbance that was neither aphasia nor dysarthria. His head CT scan was normal but the neurologist suspected an ischemic stroke and decided to hospitalize him, nonetheless. Due to the unusual presentation of the speech disturbance, a brain MRI was performed and was normal. The patient denied stress or other emotional factors, while acknowledging that he was about to receive a long-awaited promotion at work. He was ultimately discharged from the hospital with a functional diagnosis.

## Describing the Problem

These four archetypal cases are imaginary examples of patients with complex conditions who commonly present to physicians with physical complaints. Each case is different; however, in all of them patients would be best served by a bio-psycho-social conceptualization, enabling them to receive treatment from a physician and from an MHP. In today's medical milieu, this can be difficult, due to the lack of a shared paradigm. In treating the aforementioned patients, many medical professionals would adopt the following clinical strategies: In case 1, the cancer patient may give up and cease treatment for his disease. Many physicians might not realize the benefits or possess the means to refer such a patient to an MHP. In case 2, the young woman with MS will be given stronger, more effective medication which will also have stronger side effects. Once again, it may not even occur to many physicians that this patient could benefit from learning mind-body techniques, as she has a physical diagnosis. In case 3, the patient suffering from chest pain after a heart attack will continue to undergo extensive testing in an attempt to discover an organic problem. At the end of what may be a lengthy, expensive and difficult process of investigation, if no physical pathology is discovered, the physician might refer the patient to an MHP. The MHP will treat the patient based on his or her own clinical proclivities. A psychiatrist oriented toward pharmacotherapy will offer SSRIs or benzodiazepines to help the patient manage anxiety. A psychodynamic psychotherapist may give a symbolic interpretation of the physical symptoms, while an existential psychotherapist may interpret it as a fear of dying. A cognitive-behavioral therapist would likely view the patient as suffering from Health Anxiety and treat accordingly. Regardless, although it is the same individual, very different diagnostic models and ways of thinking may come into play and the patient can often become lost in the fray. In case 4, had the patient been mistakenly diagnosed with a CVA, it would likely have caused him a great deal of anxiety, he would have received inappropriate and perhaps harmful treatments and his attempts to return to work would likely have been unsuccessful and frustrating. Here too, the patient may be sent to an MHP and will generally receive treatment based on the clinical proclivities of said professional. Some may take a psychodynamic approach, while others may be more Cognitive-Behavioral Therapy (CBT)-oriented. However, both may have difficulty determining therapeutic goals. Should they be assisting the patient in learning to live with the symptoms? Should they be attempting to reduce the frequency or severity of the symptoms? Can they attempt to do away with the symptoms completely? Without a model for understanding the mind-body relationship for each case, the focus of treatment for the preceding four patients remains unclear.

## The Four-Cluster Mind-Body Model

**Figure 1:**
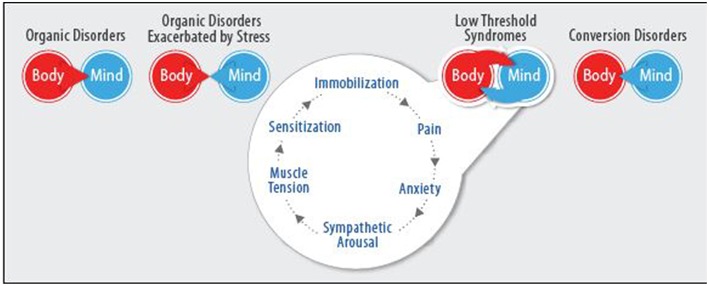
The Four-cluster model of mind-body interrelationships.

We now present a four-tiered, conceptual model that analyzes physical symptoms based on the various psycho-biological processes occurring. Each of the following clusters connotes a separate type of mind-body interaction, with diverse pathological mechanisms involved in creating symptoms, from which different treatment goals and strategies should be employed (Figure 1).

### Organic Conditions

These include structural or degenerative processes that can affect mood and psychological reactions but are not necessarily exacerbated by stress. Organic diseases should be treated primarily with conventional medical methods such as surgery, invasive interventions, pharmacotherapy or chemotherapy. Although, these conditions are not caused by psychological factors, they can catalyze psychological reactions such as anxiety and depression which may ultimately affect medical outcomes. For example, tumors or congestive heart failure can cause emotional reactions that may alter patients' decisions or health-behaviors and so influence outcomes (12, 13). In these cases, the medical treatment plan should be directed toward curing the organic disease. Psychotherapy should be directed at coping with the condition, enhancing compliance, rehabilitative therapy, or learning to live with the disability (14). One important focus of psychotherapy in this cluster is illness perceptions and behaviors. When people are diagnosed with illness, they develop an internal, common-sense set of beliefs and cognitions regarding their situation. These beliefs can be important determinants of patients' emotional response, coping patterns, adherence to treatment and other illness behaviors. Beliefs generally focus around consistent domains, such as illness identity, causation, timeline, the possibility of controlling or curing the illness and the consequences of illness. Negative illness perceptions are associated with poorer recovery and increased healthcare use. For example, patients with negative beliefs about their ability to control their illness may exhibit low levels of compliance to medical treatment (13, 15, 16). In such cases, therapeutic modalities making use of psycho-education, Cognitive Restructuring and Motivational Interviewing may be very important in improving medical outcomes (17, 18).

### Stress-Exacerbated Diseases

In this cluster, we include biological disorders that are exacerbated by stress, such as inflammatory disorders (e.g., asthma, multiple sclerosis and Crohn's disease), movement disorders (e.g., essential tremor) and pain disorders (e.g., migraine). The common factor in all these diseases is that they are primarily biological disorders with overt pathophysiology. However, due to the nature of the mind-body relationship and the ways in which emotional processes such as chronic stress can influence our physiology, they are often exacerbated by psycho-social factors (19–21).

Of particular importance to the second cluster is the aforementioned field of PNI, which describes specific biological pathways by means of which emotions, such as stress, mediate health, and disease. One pathway illuminated by PNI research is the way in which psychological factors influence the neuroendocrine system. Emotions can provoke the release of pituitary and adrenal hormones which can then affect cardiovascular, metabolic and immune functioning. Numerous studies have suggested that a variety of emotion-responsive hormones including the catecholamines (norepinephrine and epinephrine), adrenocorticotropic hormone, cortisol, growth hormone, and prolactin can impel changes in immune function (22). Studies have shown, for example, that depression can substantially boost cortisol (23) and decrease growth hormone (24), which can have adverse immunological effects. As mentioned, the immune system has an important role in mediating the effects of stress on health. Since disease occurs when host defenses are not sufficient to cope with the disease-causing agent, psychological factors that influence immunity can potentially influence the progression of many diseases. Studies have shown that high levels of stress can increase the risk for upper respiratory infections, viruses such as the flu, the common cold, and influenza. These effects remained constant, even when subjects were controlled for stress-elicited health behaviors, such as smoking and alcohol consumption. Other studies have shown connections between stress levels and susceptibility to diseases related to the herpes virus, such as cold sores and genital lesions, as well as to autoimmune diseases, such as rheumatoid arthritis, inflammatory bowel disease, lupus, and diabetes (25).

In summary, PNI emphasizes the way the hypothalamic-pituitary- adrenal (HPA) axis regulates hormonal changes, influencing the production of cytokines and white blood cells, causing either hyper-activation of the immune system in autoimmune diseases for example, or conversely, immune system hypo-activation, increasing susceptibility to viral infections (8, 22, 25). Such psycho-biological pathways are central to understanding the second cluster and help explain how pathophysiology can be affected by emotional states.

In this cluster, the mainstay of treatment should be conventional medical care, such as immunomodulating treatment for multiple sclerosis, asthma, rheumatoid arthritis and ulcerative colitis; beta blockers for tremors, and triptans for migraine. However, it is extremely important to include adjuvant behavioral treatments for stress reduction, such as relaxation training, biofeedback or hypnosis. The role of these mind-body therapies (MBT) is to reduce the effects of stress on biological pathways that influence the neuroendocrine and immune systems, thereby helping patients reduce the frequency and severity of physical symptoms caused by stress-induced exacerbation of their disease (26–28). In this manner, both types of treatment—medical and behavioral—can jointly affect the course of disease.

### Functional Somatic Syndromes (FSS) (29)

Various, sometimes loosely related appellations have been given to this third category, including Somatic Symptoms Disorders (SSD) (30), Medically Unexplained Symptoms (MUS) (31) and Central Sensitization Syndromes (CSS) (32), to name a few. These categorizations often include specific syndromes such as irritable bowel syndrome (IBS), fibromyalgia, tension-type headache (TTH) and non-cardiac chest pain (NCCP) (31). The common denominator among these various symptoms and syndromes is one or many non-specific somatic symptoms that are not wholly explicable by means of laboratory, imaging or electrophysiological investigations (33). Due to the lack of clear structural, inflammatory or degenerative processes and the high prevalence of mood and anxiety disorders associated with these conditions (34), previous bio-medical approaches have sometimes wondered whether FSS are physical conditions that cause anxiety and depression, or psychological conditions that cause physical pain (35). Currently, a significant body of medical literature points to a bi-directional, psycho-biological conditioning process whereby heightened sensitivity to stimuli combined with hyper-reactivity of the autonomic system form a “vicious cycle” of mutually enhancing bio-psychological learning processes that emanate from either the body or the mind (36–39). This vicious cycle is often preceded by trauma or excessive stress, which can dysregulate the limbic system, giving rise to psychological processes such as catastrophic misinterpretation, selective attention, fear-based conditioning and sensitization. When physical symptoms are precipitated, the efferent/autonomic pathways governed by the hypothalamus overreact, concurrently with hypersensitivity of afferent pathways controlled by the thalamus. Due to the nature of these heightened sensitivity and reactivity processes, our group has termed these syndromes, “Low Threshold Syndromes” (LTS), referring to the afferent and efferent aspects of this cycle (40). Many theories have attempted to further elucidate these biological and psychological pathways (41). We will attempt to integrate some of these ideas, so as to provide a multi-faceted model for understanding FSS.

1. **Predisposing Factors:** Predisposing factors may be genetic or the result of early gene-environment interactions, such as temperament, gender, familial influences, or adversities early in life. A modest genetic predisposition for somatic symptoms and syndromes has been demonstrated via both twin and adoption studies (42–44), while gender (45–47) and various other temperamental factors, personality traits, and early life experiences have been shown to be even more strongly correlated to the development of somatic disorders (48–54).
2. **Precipitating Factors:** Environmental factors, such as traumatic life events or extended periods of stress, can interact with an individual's predisposition, acting as catalysts in the creation of FSS. Prospective epidemiological studies have shown that various psychosocial factors, such as illness behaviors, distressing events, and anxiety are predictive of FSS (55). Studies have demonstrated relationships between FSS and psychological states such as depression (56–58) and anger (59, 60), as well as traumatic life events such as rape, abuse (61, 62), infection (63), and surgery (64).
3. **Perpetuating Factors:** Many individuals undergo stressful experiences; however, in FSS, processes set in motion by the interaction between predisposing and perpetuating factors often lead to chronic symptoms (65). In order to explain these differences, several researchers have subscribed to a transactional model based on the biopsychosocial paradigm, wherein psychological and physiological processes reinforce one another, perpetuating and aggravating symptoms by means of a “vicious cycle” effect (66–70). Eriksen and Ursin (65) proposed one such psycho-biological model which explains how FSS might begin with an initial pain or trigger, causing a state of heightened emotional arousal and attention to potentially unpleasant bodily sensations, leading to further sensitization and more pain. Thus, while predisposition may be general in nature, the chronic perpetuation of symptoms in target organs may be determined by the site and type of precipitating factor involved. A variety of biopsychosocial factors may play a role in such transactional processes and contribute to maintaining the symptoms' severity. Although a full analysis of these factors is beyond the scope of this article, we provide a concise overview of a number of salient variables:
  - Biological Factors: Neurophysiological research has found similarities in the way the brain processes sensory stimuli, such as pain, across various FSS. Studies using fMRI and PET scans have identified a network of cortical regions involved in the cognitive modulation of pain, including the anterior cingulate cortex, insula, prefrontal regions, and primary (S1) and secondary (S2) somatosensory cortices (71). Abnormal activation within this pain network may cause or partially generate functional pain disorders (38). There is evidence that such abnormal activation may begin at the level of peripheral nociception (72) and may be influenced by higher level cognitive processes, such as anxiety. Anxiety has been shown to predict pain severity and pain behavior in both acute and chronic pain patients (73). Studies have shown that stressful life events produce stronger reactions in FSS patients than in healthy controls (74). Pain modulation by anxiety is associated with activation changes in the entorhinal cortex of the hippocampal formation that interact with the perigenual cingulate and mid-insula. Hippocampal and amygdala activation are thought to modulate pain processing by amplifying signals to the areas primarily involved in pain processing (75). Various measures have been used to demonstrate heightened sensitivity to painful stimuli among FSS patients. One such measure is the nociceptive (spinal) flexion reflex (NFR), an accepted pain measure obtained by electrically stimulating the sural nerve and measuring the response of the biceps femoris. Heightened sensitivity to NFR has been shown in patients with several different somatic syndromes, as compared to healthy controls (32). An additional measure is diffuse noxious inhibitory control (DNIC), which quantitatively measures the nervous system's ability to limit the intensity of pain in response to a repeated irritant. DNIC has been found to accurately predict the development of chronic pain. The healthy mechanism whereby each pain response diminishes the intensity of the following response has been shown to be dysfunctional in patients suffering from somatic syndromes (76). Many studies have demonstrated that various pain syndromes, such as TTH, migraines, fibromyalgia (77, 78), back pain (79), neck pain (80), IBS (66), and post-operative chronic pain (81), can be tied to disruptions of DNIC pain inhibition, and that these patients have lower DNIC scores than healthy controls do.
  - Psychological Factors: Different models have indicated the influence of various patterns of thought and emotion in FSS, such as emotional repression (48, 82), alexithymia (83), catastrophic thinking (84–86), maladaptive coping strategies, external locus of control (87) and “secondary gains” (88). The common theme of many of these patterns may be that emotional and physiological over-arousal, as well as counterproductive illness behaviors engender, a cycle of psycho-biological conditioning (55, 89). This conditioning process is exacerbated by anxiously over-attenuating to and amplifying bodily sensations. As previously mentioned, changes in cognitive-sensory processing due to emotional arousal can, over time, cause sensitization of the central nervous system, so that stimuli arising in the body might be experienced as excessively painful (32, 41).
  - Behavioral Factors: In addition to interactions between biology and psychology, distressed individuals are also more likely to exhibit illness behaviors that can negatively influence their symptoms. These behaviors may include smoking, irregular sleep habits, alcohol or drug abuse, high utilization of health-care, low-adherence, poor nutrition and lack of physical exercise (22). Difficult social and interpersonal circumstances can also play a role in exacerbating detrimental behaviors and negatively influence health (90). Such behavioral and psycho-social determinants create mutually-enhancing interactions with stress and can have neurological and immunological consequences. For example, deep sleep positively affects the endocrine system, while smoking is associated with higher plasma IL-6 and CRP levels (22). Many of these behavior patterns may be equally influential in diseases belonging to the second as well as the third cluster. However, there are a number of illness behaviors that are of particular importance to the perpetuation of FSS. One important example of this is kinesiophobia. Kinesiophobia, literally “fear of movement,” refers to patients who avoid moving their bodies due to the fear of experiencing unnecessary pain. Unfortunately, in many chronic pain conditions excessive avoidance ultimately has the opposite effect of sensitizing patients even further to stimuli that may once not have been experienced as painful (91, 92). This issue is also borne out by the body of research showing that physical activity training is beneficial for many FSS, such as fibromyalgia, chronic fatigue syndrome and lower back pain (93). A second important example of counterproductive illness behaviors in FSS is forming a dependence or addiction to narcotics due to over-use of pain medication. Overuse of narcotics such as opioids can cause a decrease in pain tolerance among patients with chronic pain (94). In chronic headaches, even over-the-counter abortive pain medications can cause secondary or “medication overuse” headaches and perpetuate the cycle of pain (95, 96). Despite many newer treatment guidelines for chronic pain conditions such as fibromyalgia, which deter physicians from prescribing opioids, reported utilization of opioids remains high, as upwards of 38% of fibromyalgia patients are prescribed opioids (94).
    In summary, the interrelationship of body and mind is paramount in FSS. In these conditions, treatment should focus on physical exercise and mind-body treatments such as meditation, CBT, and hypnosis (93, 97). Pharmacotherapy should make use of selective norepinephrine reuptake inhibitors (SNRIs) or tricyclic antidepressants (TCAs) (98) and taper the use of analgesics for chronic headaches and of opioids for other forms of chronic pain, as this treatment can induce hyperalgesia (99–101). It is important to note that treatment methods that may be effective for patients in the first or second clusters, such as invasive procedures or relying primarily on pain medication, may be ineffective or even harmful with patients in the third cluster. Additionally, in contrast to the fourth cluster, symptoms should not be seen as having a symbolic role and patients' lack of insight regarding the psychological processes involved cannot be viewed primarily as representations of unconscious psychic conflict. Therefore, in the third cluster, a more direct psycho-educative approach regarding the mind-body relationship is an important therapeutic factor (40).

### Conversion Disorders (CD)

At the far end of the spectrum are symptoms related to Conversion Disorder (CD). CD has had a difficult and tumultuous history. In the past, “conversion hysteria” was often treated as a religious phenomenon. It was re-medicalized during the Renaissance and by the latter half of the nineteenth century, it was common to view CD as being caused and cured by psycho-social variables. However, even then, many neurologists viewed it as a form of malingering (102). Currently, the DSM-5 refers to CD or “Functional Neurological Disorder” purely as a condition in which there is a mismatch between presenting symptoms and recognized neurological pathology, without regard to the mechanism by which this occurs (30). In our model, CD does not necessarily refer to the phenomenon but rather to the process whereby an unconscious, intra-psychic conflict can be expressed somatically. This view is in conjunction with George Engels' discussion of CD and his assertion that: “Conversion is a psychological concept, the definition of which cannot include or be bounded by neuroanatomy, even though the function and structure of the nervous system may be involved secondarily… The parts or systems of the body capable of being involved in conversions are determined… by their capability to achieve mental representation” (103). Psychodynamic theories have provided a number of frameworks by means of which this process could take place, generally rooted in Freud's understanding that unacceptable drives can be repressed from conscious awareness and converted into physical symptoms (11). The theoretician D. W. Winnicot viewed the development of psychosomatic symptoms as the mind's attempt to cope with distress by disassociating from the body. As a child develops, natural frustrations caused by the lack of a perfectly attuned environment catalyze the creation of a “psyche” separate from the child's “soma.” If these—primarily physical—frustrations exceed the child's ability to gracefully integrate the two systems, they can be experienced as foreign and even hostile to one another, forming a self that views itself in opposition to the body (104). Joyce McDougall, another theoretician, viewed psychosomatic symptoms as physical representations of mental states. For McDougall, alexithymia—deficiency in a person's ability to create mental representations for difficult emotions—could result in those emotions being translated into physical symptoms as an unconscious form of “acting out,” quite in the same way that an angry person might slam a door instead of voicing his or her feelings (105). In such models, physical symptoms are viewed as a defense mechanism against external frustrations, used to resolve dilemmas, cope with traumatic events or escape interpersonal conflict (11). In CD, as opposed to SSD, symptoms are seen as having a psychological role, such as allowing emotional conflicts involving stress, anger or shame to be resolved via the body, without the patient having to arrive at conscious awareness of the unpleasant feelings. This generally leads to the clinical manifestation of symptoms coupled with alexithymia and low psychological insight (106). In these conditions, pharmacological and behavioral treatments will not provide a sufficient cure, as they do not deal with the psychological drama that is occurring. Rather, treatment should focus on unconscious processes and employ modalities such as hypnosis or psychodynamic therapy to help the patient achieve better resolution of the issues at the root of the symptomology.

## Discussion

After presenting these four distinct clusters, we would like to illustrate how this model can help guide treatment of the patients mentioned earlier.

The first patient has a serious medical illness. His condition will necessitate conventional medical interventions and he may additionally benefit from medically-focused psychotherapy to help him cope with his illness. This psychotherapy can be provided by a medical psychologist and include elements such as eliciting concerns, delineating motivation, conveying the potential for connection, meaning, reconciliation, and closure in the dying process (107).

The second patient has a chronic illness exacerbated by stress (108). In her case, treatment should include steroids during MS attacks and immunomodulatory medication during periods of remission. However, in addition to pharmacotherapy, psycho-physiological relaxation techniques and exercise can be effective in reducing anxiety, fatigue, bladder incontinence and daily pain intensity while improving balance and quality of life (109).

The third patient has FSS. The symptoms are caused by psycho-biological processes of stress and sensitization and do not serve any other psychic purpose in terms of the patient's mental life, as they might in the fourth cluster. Neither do they include any significant histologic or morphologic changes detectable by imaging procedures. In this case, the best course of treatment would be psycho-education regarding the nature of the physical symptoms and the mechanisms by which they are maintained, physical exercise and CBT incorporating psycho-physiological techniques such as hypnotic relaxation, meditation, or biofeedback (93, 110).

The fourth patient has Conversion Disorder. Despite denying stress as a catalyst, his symptoms can be seen as having a psychological role in preventing his return to work; thus, resolving what may be conflicting emotions about the promotion he had been about to receive. In this case, the focus of treatment must be on understanding and coming to terms with these unconscious processes via hypnoanalytic or psychodynamic psychotherapy. Stress reduction techniques will likely have little effect on the symptoms, while extensive medical investigation and pharmacotherapy will only exacerbate the condition over the long term. In our experience with such patients, resolution of the conflict, whether through psychotherapy or by external means (such as a decision to make a professional change to something better suited to his needs), will often resolve the symptoms (Table 1).

**Table 1:** Physical symptoms and their treatment stratified by the four-cluster model.

**Symptom**	**Organic conditions**	**Stress exacerbated diseases**	**Functional somatic syndromes**	**Conversion disorder**
Headaches	Secondary headaches e.g., meningitis, tumor	Migraine	Tension type headache	•
Dyspnea	Pneumonia	Asthma	Panic attack	•
Loss of consciousness	Epilepsy	Syncope	Panic attack with dissociation	Psychogenic non-epileptic seizures
Movement disorders	Parkinson's disease	Essential tremor	Tic disorder	Functional neurological syndromes
Abdominal pain	Colon cancer	Inflammatory Bowel Disease	Irritable bowel syndrome	Idiopathic abdominal pain
Chest pain	Myocardial infarction	Angina pectoris	Non-cardiac chest pain	•
Back pain	Radiculopathy		Chronic low back pain	•
Medical treatment	Standard care	Standard care	Neuro-modulatory, such as anti-depressants, anti-epileptics; no opiates, no invasive procedures	Not helpful
Psychological treatment	Coping with illness, enhancing adherence	Mind-body techniques (hypnosis, meditation, biofeedback) for stress reduction	Psycho-education, cognitive behavioral therapy, (hypnosis, meditation, biofeedback)	Psychodynamic Psychotherapy, hypnoanalysis (ego state therapy)

• *Conversion Disorders can have many different manifestations including pain, as explained in the Discussion section of this article. However, since there are not necessarily separate criteria for classifying Conversion Disorder, we left some of these categories empty*.

A number of historical and philosophical attitudes have separated mind and body, contributing to the construction of a modern medical system that incorporates little infrastructure for integrating the physical, psychological and sociological mechanisms of illness. Currently, physicians are trained to analyze symptoms based on differential diagnosis rooted in pathophysiological mechanisms. Physicians can categorize diseases as inflammatory, infectious, degenerative, malignant, genetic, metabolic, etc. These categorizations allow treatment to be tailored to the mechanism of disease; the more precise the diagnosis, the more effective the treatment. Malignant diseases can be treated with chemotherapy, inflammatory diseases with immunomodulatory treatment, infectious diseases with antibiotics and so on. Until mind-body issues are incorporated into a similar medical framework which allows for the discussion of their underlying mechanisms and delineates a clear path for treatment, it is unlikely that physicians will be able to adequately care for these patients.

Previous models of mind-body interactions have tended to fall into different camps based on particular theoretical denominations, causing seemingly contradictory approaches in many cases. Biomedical models tend to be dichotomous by nature, viewing symptoms as either physical or psychological. Most physicians will comfortably subsume patients from the first two categories of our conceptual model, often with little thought as to whether or not they might benefit from addressing psycho-social components of their condition. However, regarding the last two categories of the model, the bio-medical approach will face many difficulties. This is most clearly seen in the third cluster. For, while psychiatrists/psychologists may treat diagnosable psychopathology, patients in the third cluster, who often lack both a clear physical or mental diagnosis, fall between the cracks. This difficulty is reflected in pejorative clinical terms used for such patients, such as: “heart-sink patients” (111) and “frequent fliers” (112), and manifest across all levels of patient care, from referral and diagnosis to notification and treatment (113).

The last two categories in the model are treated in the psychiatric literature, namely, the Diagnostic & Statistical Manual (DSM-5) for mental health disorders (30). The DSM-5, as per its stated mandate, views these conditions via a purely phenomenological lens, relating not to proposed mechanisms but to symptoms. This is certainly a valid perspective, but also limiting. Two pertinent categories exist in this regard: Somatic Symptoms Disorder (SSD) and Conversion Disorder (CD), which loosely correlate with the third and fourth clusters in our model, respectively, but with some important differences. Regarding SSD, the DSM-5 emphasizes the existence of excessive thoughts, feelings or behaviors related to the symptoms. Due to this psychiatric lens, patients suffering from fibromyalgia, IBS or TTH without measurable psychological distress will be excluded, whereas in our model they would be included in the third cluster. This is significant because as demonstrated, many of the same psycho-biological mechanisms apply to such patients. Based on the same perspective, the DSM-5 criteria for diagnosing CD necessitate a clear discrepancy between somatic complaints and neurological pathophysiology. This insistence excludes pain disorders, for example, whose symptoms cannot be controverted via neurological exam, but for whom psychological processes of conversion may play a significant role. Such patients would be included in our categorization of CD.

There are, of course, models in both the psychological and medical literature that attempt to describe the mind-body relationship via specific theoretical frameworks and extrapolate therapeutic modalities. Psychodynamic models have traditionally looked at psychosomatic symptoms as the conversion of psychic pain into physiological symptoms, without discussing the biological pathways involved (2). Cognitive-behavioral models, in turn, have focused on psychosomatic symptoms as the result of psycho-biological conditioning and emphasize cognitive processes such as catastrophization (114) and negative attention to symptoms, as well as physiological and behavioral processes such as stress and sensitization (115). However, these models lack a more nuanced and systematic approach for understanding the different ways various psycho-physiological mechanisms affect the body. For example, stress affects the second and third cluster very differently. In second cluster diseases, such as Crohn's disease or MS, psychological factors, such as stress, exacerbate inflammatory processes that can be seen and measured objectively. In the third cluster, stress is involved in processes such as central sensitization but does not cause measurable histological changes. For this reason, pharmacotherapy is significantly more effective with second cluster diseases than it is with third cluster diseases.

It is our view that these varying perspectives are not contradictory but complementary and each is an appropriate description of a different group of patients. Our model provides a mechanism-driven categorization by integrating different approaches into one meta-model. Biological models are adept at explaining the first cluster, PNI studies elucidate the second, psycho-biological conditioning theories contribute to an understanding of the third, and psychodynamic theories help make sense of the fourth. It is important to note that we do not maintain that each of these domains pertains solely to one cluster, only that it provides the most concise explanation for the salient features of that cluster. PNI studies interrelate with clusters other than the second, as does psycho-biological conditioning with conditions outside of the third. However, each enables the clearest understanding of the underlying mechanisms of a particular group of conditions, allowing for more precise treatment plans, tailored to patients' needs based on an understanding of the directionality of the relationships between mind and body in each of the four clusters. The four-cluster model seeks to bridge the extant conceptual gap by creating an overarching spectrum with proposed mechanisms and directionality, by means of which therapeutic interventions may be effectively planned. A comprehensive model affords an understanding of patients' conditions by means of proposed mechanisms, as opposed to purely phenomenological perspectives. This allows theories to be placed side-by-side, so that each can inform treatment for the patients for whom they are most relevant.

Future research is needed to validate the theoretical and clinical relevance of the four-cluster model. Theoretical research should focus on forming a clearer understating of the mechanisms involved in the various clusters. In this way, our model could be disconfirmed if biological processes and matching medical treatments were to be discovered that could explain and alleviate symptoms for third and fourth cluster diseases. For example, if either biological treatment such as antibiotics could cure chronic fatigue syndrome or conversely, if psychotherapy such as hypnosis could cure cancer, our model would prove to be irrelevant.

Additionally, clinical research could compare patient outcomes when treatment is matched to the patient's position on the mind-body spectrum, as opposed to patients whose treatment is not matched. If outcome measures improve when treatments are matched to patients' placement along the four cluster spectrum, it would help support the efficacy of our conceptualization.

In summary, when physicians are aware that they must place patients along a bio-psycho-social spectrum, they can create effective clinical partnerships with MHPs and refer patients for treatment with the proper medical explanation regarding the complex interactions taking place between mind and body, resulting in the formation of discrete therapeutic goals. We believe that such partnerships would improve communication between physicians and MHPs and help clinicians avoid inappropriate treatment plans, such as using hypnosis to treat cancer or surgery to treat CD. Consultations could take place more frequently and efficiently, professional boundaries would be more clearly delineated and joint research endeavors could be undertaken with greater ease than in the past.

## Author Contributions

All authors contributed to the theoretical perspectives presented in the article. YE is credited with the original development of the four-cluster model. OH contributed extensively to further development of the model and the text of the article. GS added a great deal of insight to the model and to the way in which it interacts with other theoretical perspectives.

### Conflict of Interest Statement

The authors declare that the research was conducted in the absence of any commercial or financial relationships that could be construed as a potential conflict of interest.
